# Endothelial Function in Women with and without a History of Glucose Intolerance in Pregnancy

**DOI:** 10.1155/2013/382670

**Published:** 2013-05-29

**Authors:** Shireen Brewster, John Floras, Bernard Zinman, Ravi Retnakaran

**Affiliations:** ^1^Leadership Sinai Centre for Diabetes, Mount Sinai Hospital, Toronto, ON, Canada M5T 3L9; ^2^Division of Cardiology, Mount Sinai Hospital, Toronto, ON, Canada M5G 1X5; ^3^Division of Cardiology, University of Toronto, Toronto, ON, Canada M5S 1A1; ^4^Division of Endocrinology, University of Toronto, Toronto, ON, Canada M5S 1A1; ^5^Samuel Lunenfeld Research Institute, Mount Sinai Hospital, Toronto, ON, Canada M5G 1X5

## Abstract

*Background/Aims*. Gestational diabetes mellitus (GDM) and milder gestational impaired glucose tolerance (GIGT) identify women who are at risk of developing cardiovascular disease. Endothelial dysfunction, as indicated by impaired flow-mediated dilatation (FMD) on brachial artery ultrasound, is an early marker of vascular disease. Thus, we sought to evaluate endothelial function in women with and without recent glucose intolerance in pregnancy. *Methods*. One-hundred and seventeen women underwent oral glucose tolerance testing (OGTT) in pregnancy, enabling stratification into those with normal gestational glucose tolerance (*n* = 59) and those with GDM or GIGT (*n* = 58). 6 years postpartum, they underwent a repeat of OGTT and brachial artery FMD studies, enabling assessment of FMD and 4 secondary vascular measures: FMD after 60 seconds (FMD_60_), baseline arterial diameter, peak shear rate, and reactive hyperemia. *Results*. There were no differences between the normal gestational glucose tolerance and GDM/GIGT groups in FMD (mean 8.5 versus 9.3%, *P* = 0.61), FMD_60_ (4.1 versus 5.1%, *P* = 0.33), baseline diameter (3.4 versus 3.4 mm, *P* = 0.66), peak shear rate (262.6 versus 274.8 s^−1^, *P* = 0.32), and reactive hyperemia (576.6 versus 496.7%, *P* = 0.07). After covariate adjustment, there were still no differences between the groups. *Conclusion*. Despite their long-term cardiovascular risk, women with glucose intolerance in pregnancy do not display endothelial dysfunction 6 years postpartum.

## 1. Introduction

 Pregnancy has been described as a stress test that can identify women at risk of future chronic disease [1]. One such high-risk population identified in pregnancy consists of women who develop gestational diabetes mellitus (GDM), defined as glucose intolerance of varying severity with first onset or recognition in pregnancy [2]. Although most women return to normoglycemia in the early postpartum period, women with a history of GDM have an increased risk of subsequently developing prediabetes and type 2 diabetes (T2DM) in the years thereafter [3, 4]. Besides dysglycemia, women with GDM exhibit other elements of an enhanced cardiovascular risk factor profile by as early as 3 months postpartum, including both (i) traditional risk factors, such as hypertension, dyslipidemia, and metabolic syndrome [5, 6], and (ii) nontraditional risk factors, such as increased C-reactive protein and low circulating levels of the fat-derived protein adiponectin [7]. Indeed, despite their relative youth (i.e., childbearing age), women with GDM have a 70% higher incidence of cardiovascular disease (CVD) as compared to their peers, within just 11 years following the index pregnancy [8]. Furthermore, even milder gestational impaired glucose tolerance (GIGT) is associated with an increased risk of subsequent T2DM, an enhanced cardiovascular risk factor profile, and ultimately a higher incidence of CVD 12 years postpartum [4–6, 9]. It thus appears that the diagnosis of glucose intolerance in pregnancy identifies a population of young women who are at risk of developing vascular disease later in life [10–12].

The development of endothelial dysfunction is an early pathologic event in the natural history of CVD. Endothelial function can be assessed noninvasively using the ultrasound-based technique of flow-mediated dilatation (FMD) [13], in which inflation of a blood pressure cuff downstream of the vessel under study produces a stimulus for endothelial production of vasoactive autocoids (such as nitric oxide) that leads to upstream arterial dilatation [14]. Impaired FMD has been associated with both cardiovascular risk factors [13, 15–17] and the presence of coronary artery disease at angiography [18]. Furthermore, impaired FMD has been shown to predict incident of CVD in certain populations [19, 20]. In this context, we hypothesized that women with previous gestational glucose intolerance may show evidence of impaired endothelial function, when compared to women who maintain normal glucose tolerance in pregnancy. To date, however, the few studies of FMD in relation to gestational glucose tolerance have yielded conflicting results, possibly owing to their modest sample sizes (ranging from 34 to 52 women) and varying degrees of adjustment for potential confounders [21–23]. Thus, our objective in the current study was to evaluate endothelial function through the assessment of FMD in a large, well-characterized cohort of women with and without a recent history of glucose intolerance in pregnancy. 

## 2. Methods

### 2.1. Subjects

This study was conducted in the setting of an ongoing research program, in which pregnant women are recruited at the time of GDM screening and then followed longitudinally in the years thereafter. For this study, women were recruited in late second or early third trimester of pregnancy and all underwent a 3-hour, 100 g oral glucose tolerance test (OGTT) for ascertainment of gestational glucose tolerance status. This OGTT classified gestational glucose tolerance status as either (i) GDM (as defined by 2 or more glucose values on the OGTT meeting National Diabetes Data Group (NDDG) criteria [24]), (ii) GIGT (as defined by only 1 glucose value meeting NDDG criteria), or (iii) normal glucose tolerance (NGT) (no glucose values meeting NDDG criteria). At ~6 years postpartum, participants underwent a 2-hr 75 g OGTT testing (on which current glucose tolerance status was classified as either diabetes, prediabetes, or normal glucose tolerance, according to Canadian Diabetes Association Clinical Practice Guidelines [25]) and brachial artery FMD studies. This study complies with the Declaration of Helsinki, was approved by the Mount Sinai Hospital Research Ethics Board, and all participants provided written informed consent. 

### 2.2. Vascular Studies

#### 2.2.1. Subject Preparation

Subject preparation for brachial artery FMD studies followed established guidelines [26]. All subjects were studied after abstaining from alcohol, cigarette smoking, exercising for 12 hours, and after an overnight fast (minimum of 8 hours). In addition, subjects were asked to abstain from vitamins for 24–48 hours. An attempt was made to perform these studies in the follicular phase of the menstrual cycle, if possible.

#### 2.2.2. FMD Protocol

On the morning of the vascular study, participants completed an interviewer-administered questionnaire that addressed personal and family medical history, use of medications (including vitamins and contraceptives), smoking history, physical activity, and menstrual patterns. Height and weight were measured by medical scale. Anatomical waist, iliac waist, and trochanteric hip were each measured twice with mean values calculated for analyses.

**Figure 1:**
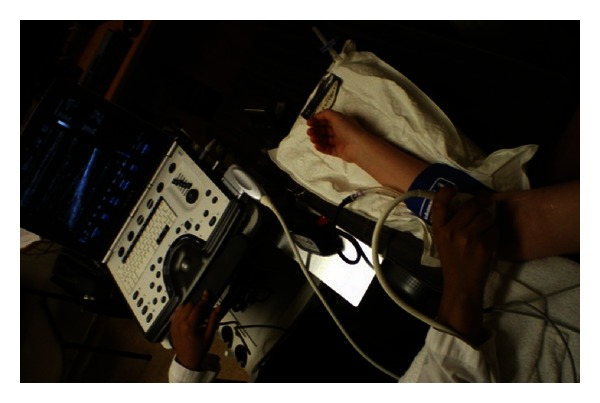
Setup of FMD studies showing proximal manual probe holding technique and distal cuff position.

All FMD studies were conducted between 07:00–11:00 AM in a quiet, dimly lit, temperature-controlled room (24–26°C). Sitting blood pressure (BP) was measured from the left arm (Baumanometer wall unit 33, W.A. Baum Co Inc., Copiague, NY) at the beginning of the test. Subjects then lay supine and the left arm BP was again recorded with pulse rate. After >10 minutes of rest, ECG electrodes (Medi-Trace 530 Series, Ludlow Technical Products Ltd., Gananoque, ON, Canada) were attached. A Versa form Pillow (12 × 22′′ Tumbleforms, Sammons, Preston, IL) was used for right arm stabilization, with arm below heart level and at an angle of 80–90° of the arm to the body. A rapid deflating manual cuff (Hokanson SC5, Bellevue, Seattle, WA) was placed 4.5 cm below the olecranon process, with the 12 MHz linear array ultrasound probe (GE Healthcare, Mississauga, ON, Canada) held proximal to the elbow (Figure 1).

 All brachial artery imaging were performed using a non-ECG gated Vivid Q GE ultrasound machine (GE Healthcare, Mississauga, ON, Canada), with manual probe placement by an experienced ultrasonographer who had performed >100 brachial ultrasound scans prior to this study. The ultrasonographer was blinded to gestational and current glucose tolerance status of the participants. All scans were continuous, employing an insonation angle of <60° and captured both brachial artery diameter and velocity measurements. Three separate scans were recorded per subject. A resting scan captured 1 minute of baseline brachial artery diameter and velocity data. Then, the cuff was inflated (Hokanson DS400 manual cuff inflator, Bellevue, Seattle, WA) to systolic BP >200 mmHg for 5 minutes. Scanning was then resumed in the final 10–20 seconds prior to cuff deflation and continued for 200 seconds. The final recovery scan captured the last 2 minutes of data. All data were stored using B-mode ultrasound on the echocardiography machine, resulting in a 15-second interruption in data acquisition during storage. After all images were obtained, supine and sitting BP were again recorded, and the average of pre- and poststudy data measurements was determined. 

#### 2.2.3. Vascular Function Outcomes

The following five measures of vascular function were assessed.

- FMD of the brachial artery was the primary vascular function outcome of interest. FMD in response to the hyperemia that occurs following cuff deflation reflects endothelium-dependent vasodilation. Specifically, hyperemia provides a mechanical stimulus (shear stress) for increased endothelial production of nitric oxide, which in turn diffuses into the underlying vascular smooth muscle cells and stimulates their production of cyclic guanosine monophosphate, which leads to vasodilation.
- Historically, FMD has been measured between 45 and 60 seconds after cuff deflation, and an impaired FMD at this time, that is, ~FMD_60_ is also a marker of CVD risk [13].
- Peak shear rate is a surrogate of peak shear stress [27] which is the stimulus for the endothelial production of nitric oxide that drives FMD [14]. A reduced peak shear stress stimulus is associated with reduced FMD [28].
- Reactive hyperemia measures the downstream dilatation of the microvasculature in response to ischemia and generates the shear stress stimulus for FMD [29].
- Brachial artery diameter is a determinant of FMD [30] and has itself been associated with cardiovascular risk factors [31].

#### 2.2.4. Analysis of Vascular Function Measurements

**Figure 2:**
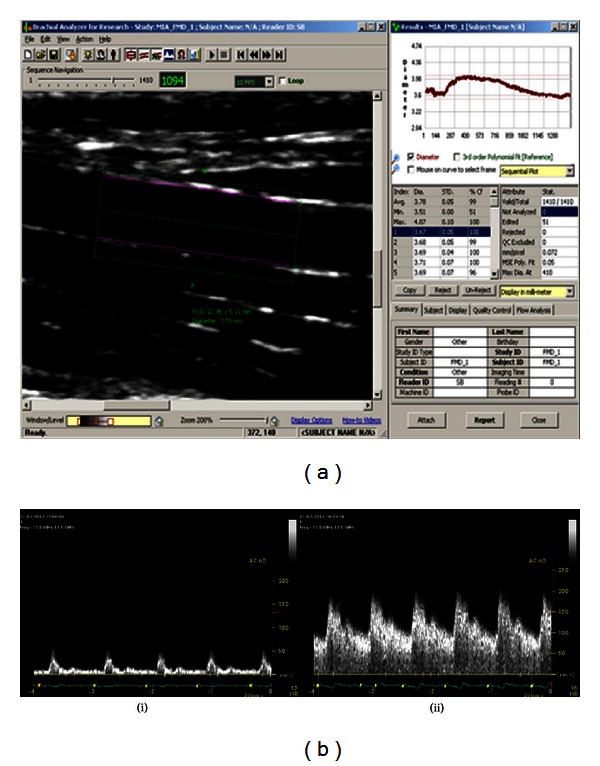
(a) Image of a longitudinal view of a brachial artery analyzed by customized edge detection software producing diameter measurements. (b) Velocity data for the brachial artery at (i) rest and (ii) hyperemia on cuff deflation.

Following image acquisition and storage, analysis of the brachial artery diameter was performed using near- and far-wall detection by customized edge detection software by Medical Imaging Applications (MI.A Vascular Tools 5, Brachial Analyzer, Coralville, Iowa) (Figure 2). All analyzed scans had a frame rate of 7-8 frames/second. Baseline or resting diameter was calculated as the average diameter of all analyzed frames obtained over the first 60 seconds. The hyperemic diameter was calculated as the average diameter of the first 15 analyzed frames after cuff deflation. FMD was expressed as a percentage and calculated by the following formula:

(1) $$\begin{array}{c} \text{FMD}=\left(\frac{{\text{diameter}}_{\text{peak}}-{\text{diameter}}_{\text{baseline}}}{{\text{diameter}}_{\text{baseline}}}\right)\times 100\%, \end{array}$$

(see [13]).

FMD_60_ was calculated using the formula:

(2) $$\begin{array}{c} {\text{FMD}}_{60}=\;\left(\frac{D_{55\text{–}65}-D_{\text{baseline}}\;}{D_{\text{baseline}}}\right)\times 100\%, \end{array}$$

where *D*_55–65_ was the average diameter in the 55–65 seconds after cuff deflation.

 The velocity measurements were calculated manually from the time-velocity integral software of the Vivid Q machine. For the rest scans, the average of 4 traced velocity envelopes was used to obtain the time-averaged mean (TAmean_rest_) velocity. For the hyperemic phase, the first envelope after cuff deflation was omitted, and the next 15 envelopes were traced. The average of these values was calculated and used as the TAmean_hyperemia_. 

The blood flow (BF) at rest and hyperemia was calculated using the following formula: 

(3) $$\begin{array}{c} \text{blood}\;\;\text{flow}=\left(\pi \left[\frac{D^{2}}{4}\right]\times V\times 60\right), \end{array}$$

(see [32]), where *D* and *V* are the corresponding diameter and TAmean velocity measurements. 

Reactive hyperemia (RH) was calculated by the following formula:

(4) $$\begin{array}{c} \text{RH}=\left(\frac{{\text{BF}}_{\text{hyperemia}}-{\text{BF}}_{\text{rest}}}{{\text{BF}}_{\text{rest}}}\right)\times 100\%, \end{array}$$

(see [13]), where BF_hyperemia_ and BF_rest_ correspond to BF during the hyperemic and rest phases of the scan.

Shear rate was calculated according to the following formula:

(5) $$\begin{array}{c} \text{shear}\;\;\text{rate}=\frac{V}{D}, \end{array}$$

(see [33]), where *V* was the TAmean velocity, and *D* was the average MIA diameter. 

#### 2.2.5. Reproducibility of Measures

In our laboratory, the interobserver coefficient of variation for FMD was 20.3% and the intraobserver coefficient of variation was 11.2%. These were calculated as the difference between the paired values divided by the mean and divided by $\sqrt{2}$ [34] on data from 12 subjects. The intraobserver and interobserver intraclass correlation coefficients for FMD were 0.9 and 0.86, respectively. The inter- and intraobserver coefficients of variation were (i) 1.4% and 1.42% for baseline diameter and (ii) 2.58% and 1.7% for peak diameter, respectively. 

### 2.3. Statistical Analysis

All analyses were conducted using SAS version 9.1 (SAS Institute, Cary, NC). Continuous variables were tested for normality of distribution, and transformations of skewed variables were used, where necessary, in subsequent analyses. The study participants were stratified into 2 groups based on their glucose tolerance status in pregnancy: (i) those with normal glucose tolerance in pregnancy and (ii) those with GDM or GIGT. Univariate differences between the 2 groups were assessed by Wilcoxon two-sample test for continuous variables and *χ* ^2^ test or Fischer's exact test for categorical variables. For each of the five vascular outcome measures (FMD, FMD_60_, baseline diameter, peak shear rate, and reactive hyperemia), adjusted mean levels were compared between the 2 groups by analysis of covariance, with adjustment for the following factors: age, years since delivery, ethnicity, body mass index (BMI), supine diastolic blood pressure (DBP), current glucose intolerance, cigarette smoking in the last month, and menstrual cycle status (follicular versus luteal). Area under the glucose curve (AUC_gluc_) on the OGTT was calculated using the trapezoidal rule and provided a continuous measure of current glycemia, in addition to categorical glucose tolerance status. A *P* value of <0.05 indicated statistical significance.

## 3. Results

### 3.1. Characteristics of Study Population

**Figure 3:**
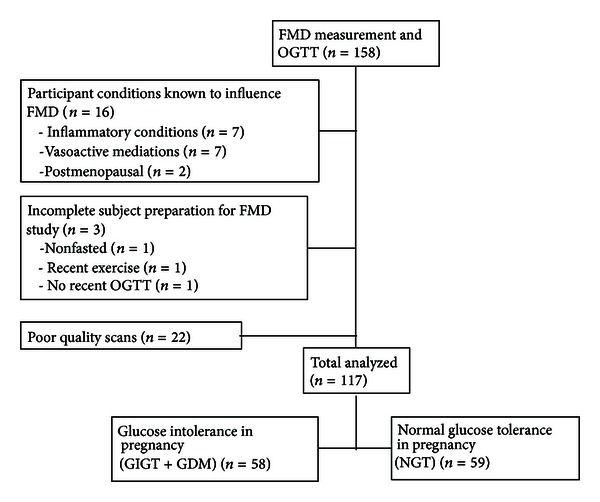
Schematic showing disposition of the study population.

Brachial artery FMD studies were performed on 158 women between May 2011 and June 2012 (Figure 3). Of this total, 41 scans were excluded because of comorbid conditions or medications that could potentially confound FMD (*n* = 16), inappropriate participant preparation prior to the study (*n* = 3), or poor quality scans (*n* = 22). Thus, the study population for analysis consisted of 117 women, who were stratified into 2 groups based on their glucose tolerance status in pregnancy: (i) NGT (*n* = 59) and (ii) GDM/GIGT (*n* = 58, consisting of 38 with GDM and 20 with GIGT).

The mean time since the index pregnancy was ~6 years for both groups (Table 1). Compared to the women who were normoglycemic in pregnancy, the GDM/GIGT group had higher sitting and supine DBP (*P* = 0.03 and *P* = 0.04, resp.) and higher AUC_gluc_ on their recent OGTT (*P* = 0.01), although the prevalence of prediabetes/diabetes did not differ between the groups (*P* = 0.28). Otherwise, there were no significant differences between the groups in age, ethnicity, anthropometry, smoking, menstrual cycle, or use of contraception.

### 3.2. Vascular Function Measures

On unadjusted comparison (Table 1), there were no significant differences between the NGT and GDM/GIGT groups in FMD (mean 8.5 versus 9.3%, *P* = 0.61), FMD_60_ (4.1 versus 5.1%, *P* = 0.33), baseline diameter (3.4 versus 3.4 mm, *P* = 0.66), peak shear rate (262.6 versus 274.8 s^−1^, *P* = 0.32), and reactive hyperemia (576.6 versus 496.7%, *P* = 0.07). Furthermore, the adjusted mean FMD also did not differ between the two groups after adjustment for age, years since delivery, ethnicity, current BMI, supine DBP, current glucose intolerance, smoking history, and menstrual status (Table 2). Similarly, there was no significant difference upon adjustment for AUC_gluc_ in place of the current categorical glucose intolerance (data not shown). In addition, there were no differences between the two groups in adjusted mean levels of FMD_60_, baseline diameter, peak shear rate, or reactive hyperemia (Table 2).

The unadjusted and adjusted comparisons of all 5 vascular measures were repeated after further stratifying the GDM/GIGT group into the 38 women with GDM and the 20 women with GIGT. Again, with this approach, there were no significant differences between the NGT, GIGT, and GDM groups in FMD, FMD_60_, baseline diameter, peak shear rate, and reactive hyperemia (data not shown).

Finally, multiple linear regression analyses were performed to identify independent determinants of the vascular function measures amongst the following covariates: age, gestational glucose intolerance, years since delivery, ethnicity, current BMI, supine DBP, current glucose intolerance, smoking, and menstrual cycle status. On these analyses, Caucasian ethnicity emerged as a negative predictor of baseline diameter (*t* = −2.65, *P* = 0.009), and BMI was a negative independent determinant of reactive hyperemia (*t* = −2.59, *P* = 0.01).

### 3.3. Post Hoc Power Calculation

 In light of the absence of differences in FMD between the NGT and GIGT/GDM groups, we performed post hoc power calculations based on the sample size. These calculations showed that a sample size of 117 participants would provide 82% power to declare no difference in FMD between study groups at a 5% significance level, under the assumption of 2.1% as the largest clinically significant difference in FMD between groups. Given the observed difference of FMD between the two groups of 0.8 and standard deviation of 3.8 with the sample size of *n* = 117, we can conclude that there was no difference of FMD between the study groups. 

## 4. Discussion

In this study, we demonstrate that, when assessed at 6 years postpartum, women with previous gestational dysglycemia exhibit similar FMD to that seen in women who maintained normal glucose tolerance in pregnancy. Furthermore, these two groups of women did not differ with respect to other vascular function measures, including FMD_60_, baseline arterial diameter, peak shear rate, and reactive hyperemia. It thus appears that, despite their long-term cardiovascular risk, women with glucose intolerance in pregnancy do not show evidence of endothelial dysfunction at 6 years postpartum.

Previous studies of FMD in women with a history of gestational dysglycemia have yielded inconsistent findings. In a study of 40 women assessed at 2 months postpartum, Davenport et al. found that brachial artery FMD was lower in women with previous GDM (*n* = 20), as compared to controls [22]. Similarly, when Anastasiou and colleagues compared obese women with GDM (*n* = 16), lean women with GDM (*n* = 17), and lean controls (*n* = 19) at 3–6 months after delivery, they noted that women with GDM had lower FMD [21]. In contrast, in a study of 17 women with GDM and 17 controls assessed at 5 years postpartum, Hannemann et al. found no difference in FMD between the two patient groups [23]. These conflicting findings may reflect the effect of several limitations that apply broadly to these studies. First, the sample sizes in these studies were quite modest, with the largest involving only 52 women. Second, there was limited characterization of other factors that could confound the relationship between previous gestational glucose intolerance and endothelial function. For example, phase of the menstrual cycle, smoking, and recent vigorous exercise [26], as well as oral contraceptives [35] are all known to influence FMD and thus need to be considered in these analyses. Third, covariate adjustment for potential confounding differences was not always performed. For example, in the study by Anastasiou and colleagues [21], the obese GDM group was older and had higher systolic and diastolic BP than did the comparator groups. These limitations may have contributed to the discordant findings reported to date.

In this context, the current study was specifically designed to address this unresolved question of endothelial function in women with previous gestational dysglycemia, while accounting for these limitations. First, the sample size of this study is more than 2-fold greater than that of the previous reports. Second, participants underwent detailed clinical characterization, including prospective ascertainment of glucose tolerance status both during pregnancy and again when undergoing the vascular studies ~6 years later. Moreover, this phenotypic detail enabled application of strict exclusion criteria that were designed to limit the influence of confounding factors (e.g., as shown in Figure 3, medical conditions and medications that could affect FMD and inappropriate participant preparation prior to vascular testing were exclusion criteria applied to the derivation of the study population). Third, in addition to unadjusted comparisons, we performed adjusted analyses to account for the effects of both observed differences between the study groups (such as diastolic BP) and potentially important covariates (such as age, years since delivery, ethnicity, BMI, current glycemia, smoking, and menstrual cycle). Finally, the participants underwent comprehensive testing of 5 vascular function measures with an experienced ultrasonographer who was blinded to both gestational and current glycemic statuses. 

 Supported by these strengths in design, the current study demonstrates that women with and without previous glucose intolerance in pregnancy have similar FMD, FMD_60_, baseline arterial diameter, peak shear rate, and reactive hyperemia at 6 years postpartum. This FMD result supports the findings of Hannemann and colleagues at 5 years postpartum, but differs from the two other reports at 2–6 months after delivery. We believe that it is unlikely that time since delivery is a direct determinant of endothelial function, particularly since this covariate was not independently associated with any of the vascular function outcomes on multiple linear regression analyses. However, it remains possible that a factor specific to the early postpartum could result in abnormalities in FMD in patients with GDM. One such consideration would be breastfeeding, which has been associated with reduced long-term cardiovascular risk [36, 37] and is less prevalent in women with GDM [38]. Irrespective, we can conclude from our findings that women with gestational dysglycemia do not exhibit endothelial dysfunction by 6 years postpartum.

 In addition, the clinical implications of these data are that FMD measurement may not be a sensitive or useful method for identifying those women with previous glucose intolerance who are at the highest risk of future CVD. Unlike in men [18], the role of FMD in CVD risk prediction has not been determined in 40-year-old women. As well, recent evidence has challenged the predictive value of FMD over established risk models such as the Framingham Risk score (FRS). In a large metaregression analysis of 399 populations where >11,000 patients were stratified by tertile of FRS, an inverse relationship between FMD and FRS was confirmed for low-risk populations only [39]. In populations of intermediate or high Framingham risk, relationships with FMD were either weak or absent. Similarly, a recent study evaluating the added prognostic capacity of novel risk factors over the FRS in 1330 patients found that the association between FMD and risk of CVD was attenuated after adjustment for confounding variables [40]. Furthermore, amongst those patients who sustained a CVD event over 7.5 years of followup, FMD did not appropriately reclassify moderate-risk subjects into the high-risk group. Thus, in light of these data, it may not be surprising that, despite their long-term risk of CVD, women with gestational dysglycemia do not show differences in FMD when compared to their peers 6 years later.

 A limitation of this study is that the assessment of vascular function at a single point in time (i.e., 6 years postpartum) does not provide insight on the potential for differential longitudinal changes in endothelial function that may emerge over time in the study groups. Similarly, the effect over time of incident prediabetes/diabetes on the relationship between gestational dysglycemia and vascular function also remains to be established. Indeed, on multiple linear regression analyses, current glucose intolerance nearly reached statistical significance as an independent negative predictor of FMD_60_ (*t* = −1.96, *P* = 0.053) (data not shown). It remains to be seen if the development of prediabetes (i) precedes endothelial dysfunction, (ii) occurs concurrently with vascular dysfunction, or (iii) is unrelated. Further longitudinal study of the temporal relationship between glycemia and vascular function is warranted in this patient population and may provide insight on the early natural history of CVD in young women.

In summary, at 6 years postpartum, women with previous glucose intolerance in pregnancy do not exhibit impairment of FMD as compared to their peers. Furthermore, these two groups of women also do not differ with respect to other measures of vascular function, including FMD_60_, baseline arterial diameter, peak shear rate, and reactive hyperemia. Thus, despite their long-term cardiovascular risk, women with glucose intolerance in pregnancy do not show evidence of endothelial dysfunction 6 years later. Future investigation should be directed at identification and validation of alternate biomarkers of subsequent vascular risk that may be evident in such women before the development of diabetes, metabolic syndrome, or cardiovascular events. 

## Figures and Tables

**Table 1:** Characteristics of study population stratified by glucose tolerance status in pregnancy.

Characteristic	Gestational glucose tolerance status		*P*
	NGT (*n* = 59)	GIGT/GDM (*n* = 58)	
Age (years)	41.1 ± 4.6	41.1 ± 4.4	0.91
Caucasian/noncaucasian	47/12	44/14	0.62
Time from delivery to FMD study (years)	5.9 ± 1.1	6.1 ± 1.1	0.59
Time from delivery to OGTT (years)	5.6 ± 1.2	5.5 ± 1.2	0.81
Body mass index (kg/m^2^)	25.8 ± 5.2	27.2 ± 5.4	0.12
Waist: hip ratio	0.82 ± 0.06	0.83 ± 0.06	0.37
Oral glucose tolerance test:			
Fasting glucose (mmol/L)	4.6 ± 0.4	4.8 ± 0.6	0.13
2-hour glucose (mmol/L)	7.7 ± 1.4	8.3 ± 1.6	0.05
AUC_gluc_	13 ± 3.1	14.7 ± 3.6	0.01
Prediabetes or diabetes (*n*)	14	19	0.28
Mean seated systolic BP (mmHg)	108.4 ± 11.6	110.6 ± 14.8	0.50
Mean supine systolic BP (mmHg)	92.6 ± 13.2	95.6 ± 12.6	0.15
Mean seated diastolic BP (mmHg)	69.7 ± 9.8	73.8 ± 10.4	0.03
Mean supine diastolic BP (mmHg)	58.6 ± 10	62 ± 9	0.04
Heart rate (beats per minute)	62.6 ± 6.7	64.5 ± 9	0.31
Menstrual state (Follicular/Luteal)	39/18	37/16	0.87
Oral contraception (N/Y)	53/6	48/10	0.27
Smoker (N/Y)	57/2	54/4	0.39
Flow-mediated dilatation %	8.5 ± 3.8	9.3 ± 3.8	0.61
Flow-mediated dilatation_60 _%	4.1 ± 4	5.1 ± 4.1	0.33
Baseline diameter (mm)	3.4 ± 0.4	3.4 ± 0.4	0.66
Peak shear rate (s^−1^)	262.6 ± 86.7	274.8 ± 74.8	0.32
Reactive hyperemia %	576.6 ± 256.2	496.7 ± 266.7	0.07

Data are presented as mean ± standard deviation. N: no, Y: yes.

**Table 2:** Mean adjusted levels of vascular measures from gestational glucose tolerance group after adjustment for age, years since delivery, ethnicity, current BMI, mean supine DBP, current glucose intolerance, smoking history, and menstrual status.

Vascular measure	Gestational glucose tolerance status		*P*
	NGT (*n* = 59)	GIGT/GDM (*n* = 58)	
Flow-mediated dilatation (%)	7.6 ± 1.1	8.7 ± 1.1	0.19
Flow-mediated dilatation_60 _(%)	2.4 ± 1.1	3.8 ± 1.1	0.09
Baseline diameter (mm)	3.4 ± 0.1	3.5 ± 0.1	0.60
Peak shear rate (s^−1^)	272 ± 22	276 ± 21	0.80
Reactive hyperemia (%)	531 ± 71	458 ± 63	0.14

Adjusted data are presented as mean ± standard error.
